# A triclinic polymorph of bis­(μ-di-*tert*-butyl­phosphanido)bis­[(di-*tert*-butyl­phosphane)palladium(I)]

**DOI:** 10.1107/S1600536812023574

**Published:** 2012-05-31

**Authors:** Jens Breunig, Hans-Wolfram Lerner, Michael Bolte

**Affiliations:** a Institut für Anorganische und Analytische Chemie, Goethe-Universität Frankfurt, Max-von-Laue-Strasse 7, 60438 Frankfurt am Main, Germany

## Abstract

A new polymorph of the title compound, [Pd_2_(C_8_H_18_P)_2_(C_8_H_19_P)_2_], has been found. It belongs to the triclinic *P*-1 space group, whereas the known form [Leoni, Sommovigo, Pasquali, Sabatino & Braga (1992 ▶), *J. Organo­met. Chem.* **423**, 263–270] crystallizes in the monoclinic *C*2/*c* space group. The title compound features a dinuclear palladium complex with a planar central Pd_2_(μ-P)_2_ core (r.m.s. deviation = 0.003 Å). The Pd—Pd distance of 2.5988 (5) Å is within the range of a Pd^I^—Pd^I^ bond. The mol­ecules of both polymorphs are located on a crystallographic centre of inversion. The mol­ecular conformations of the two polymorphs are essentially identical. The crystal packing patterns, on the other hand, are slightly different.

## Related literature

For synthetic background, see: Dornhaus *et al.* (2006*a* ▶,*b* ▶); Kückmann *et al.* (2005 ▶); Lerner (2005 ▶); Sänger *et al.* (2012 ▶). For the monoclinic polymorph of the title compound, see: Leoni *et al.* (1992 ▶). For the Cambridge Structural Database, see: Allen (2002 ▶).
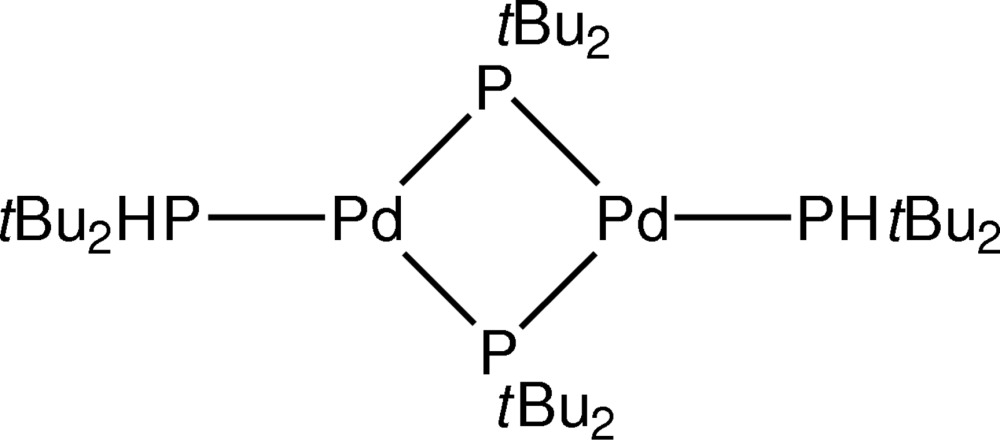


## Experimental

### 

#### Crystal data

[Pd_2_(C_8_H_18_P)_2_(C_8_H_19_P)_2_]
*M* *_r_* = 795.59
Triclinic, 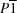

*a* = 9.0721 (5) Å
*b* = 10.5156 (6) Å
*c* = 11.5351 (6) Å
α = 89.064 (5)°
β = 67.307 (4)°
γ = 75.330 (5)°
*V* = 978.06 (9) Å^3^
*Z* = 1
Mo *K*α radiation
μ = 1.10 mm^−1^
*T* = 173 K
0.23 × 0.14 × 0.04 mm

#### Data collection

Stoe IPDS II two-circle diffractometer
Absorption correction: multi-scan (*MULABS*; Spek, 2009 ▶; Blessing, 1995 ▶) *T*_min_ = 0.786, *T*_max_ = 0.957
13922 measured reflections
4481 independent reflections
4083 reflections with *I* > 2σ(*I*)
*R*_int_ = 0.066

#### Refinement

*R*[*F* ^2^ > 2σ(*F* ^2^)] = 0.042
*wR*(*F* ^2^) = 0.111
*S* = 1.05
4481 reflections
176 parameters
H atoms treated by a mixture of independent and constrained refinement
Δρ_max_ = 2.31 e Å^−3^
Δρ_min_ = −1.34 e Å^−3^

### 

Data collection: *X-AREA* (Stoe & Cie, 2001 ▶); cell refinement: *X-AREA*; data reduction: *X-AREA*; program(s) used to solve structure: *SHELXS97* (Sheldrick, 2008 ▶); program(s) used to refine structure: *SHELXL97* (Sheldrick, 2008 ▶); molecular graphics: *XP* (Sheldrick, 2008 ▶); software used to prepare material for publication: *SHELXL97*.

## Supplementary Material

Crystal structure: contains datablock(s) I, global. DOI: 10.1107/S1600536812023574/ng5274sup1.cif

Structure factors: contains datablock(s) I. DOI: 10.1107/S1600536812023574/ng5274Isup2.hkl

Additional supplementary materials:  crystallographic information; 3D view; checkCIF report

## Figures and Tables

**Fig. 1.**
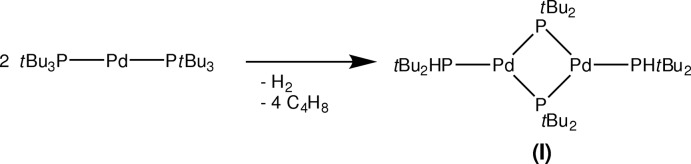
Reaction scheme for obtaining the title compound.

**Fig. 2.**
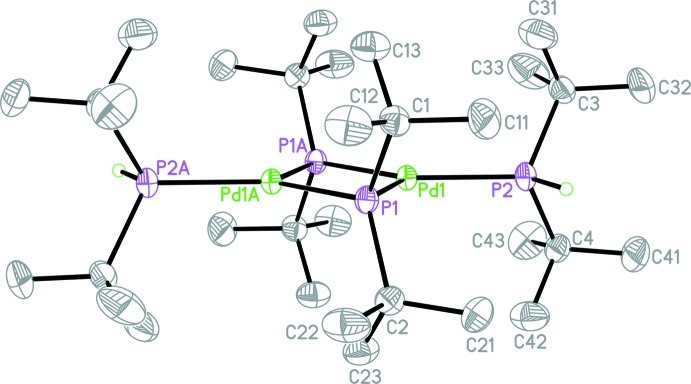
A perspective view of the title compound, showing the atom-numbering scheme. Displacement ellipsoids are drawn at the 50% probability level. H atoms bonded to C are omitted for clarity. Atoms with the suffix A were generated by the symmetry operator -x + 1, -y, -z + 1.

**Fig. 3.**
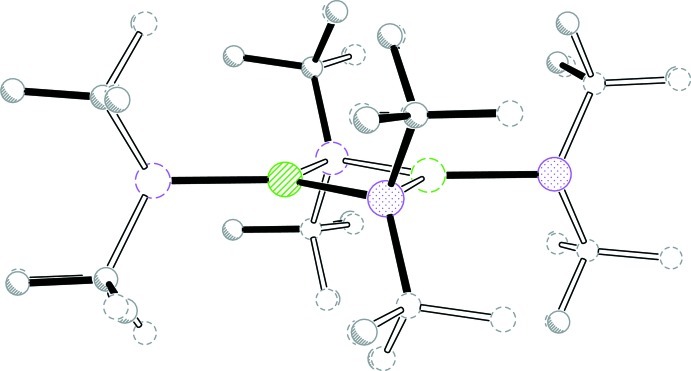
Least-squares fit of the two polymorphs of the title compound. The triclinic polymorph is drawn with full bonds, the monoclinic form with open bonds.

**Fig. 4.**
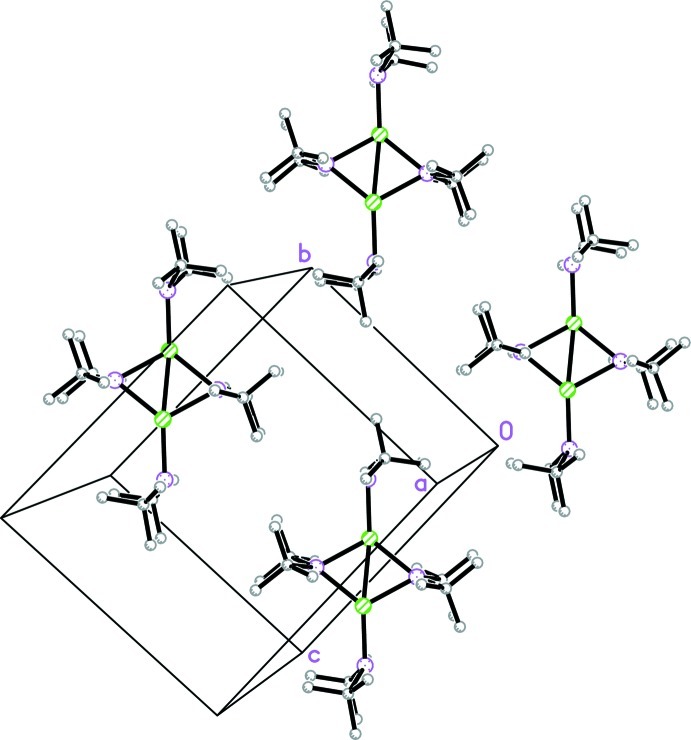
Packing diagram of the triclinic polymorph of the title compound.

**Fig. 5.**
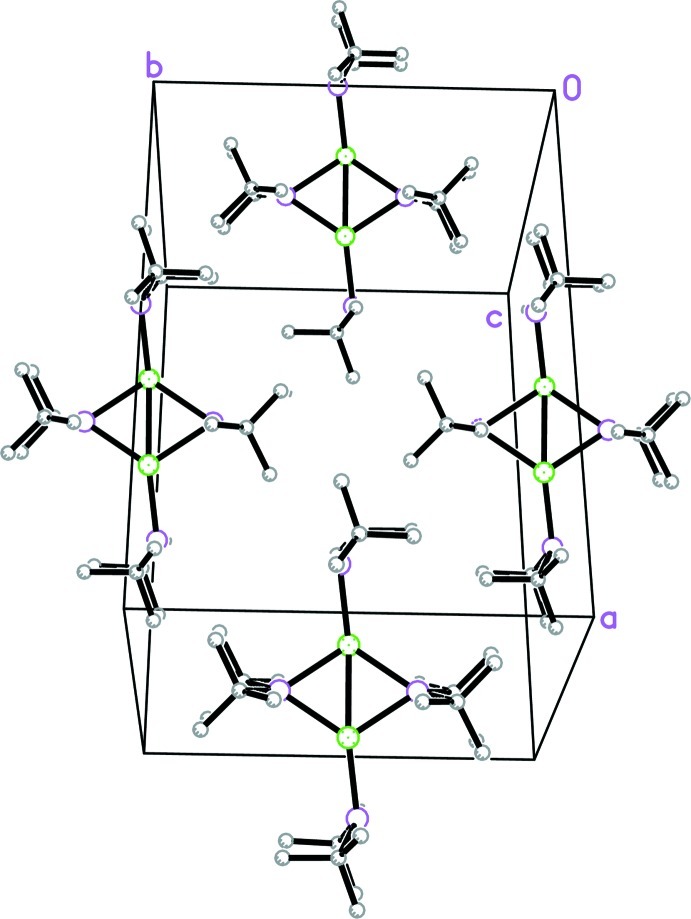
Packing diagram of the monoclinic polymorph of the title compound.

## supplementary crystallographic information

### Comment

Recently, we have made a comparison of cyclopentadienyliron dicarbonyl complexes with two series of isoelectronic and largely isosteric ligands (Sänger *et al.*, 2012), namely phosphanes P*R*_3_, phosphanyl borohydrides [P*R*_2_BH_3_]^-^ (Dornhaus *et al.*, 2006*a*), and silanides [Si*R*_3_]^-^ (Lerner, 2005), and the corresponding chalcogene derivatives SP*t*Bu_3_, [SP*t*Bu_2_BH_3_]^-^ (Dornhaus *et al.*, 2006*b*) and [SSi*t*Bu_3_]^-^ (Kückmann *et al.*, 2005). We have concluded that, with respect to electron donor strength, phosphanyl borohydrides occupy an intermediate position between phosphanes (weakest donors) and silyl ligands (strongest donors). In addition we found that electron-rich *t*Bu-substituted silanides tend to liberate isobutene. In this paper we report the structure of a new polymorph of [(*t*Bu_2_HP)PdP*t*Bu_2_]_2_, (I). The title compound was obtained *via* photolysis of Pd(P*t*Bu_3_)_2_ accompanied by liberation of isobutene and dihydrogen (Fig. 1).

A perspective view of the title compound is shown in Fig. 2. The molecules lie on a crystallographic centre of inversion. In the monoclinic form, the molecules also show *C_i_* symmetry. A least-squares fit of the P and Pd atoms (r.m.s. deviation 0.004 Å) of the two polymorphs shows that the two structures are essentially identical (Fig. 3). The crystal packing patterns, on the other hand, are slightly different (Figs. 4 and 5). The value of the acyclic P—Pd bond [2.2860 (8) Å] agrees well with 2.29 (4) Å, which was found for comparable structures in the Cambridge Structural Database (Version 5.33 of November 2011, plus one update; Allen, 2002). The cyclic P—Pd bonds [2.3374 (9) Å, 2.3405 (8) Å] and the Pd···Pd [2.5988 (5) Å] distance, on the other hand, are slightly longer than the database values [P—Pd = 2.315 (16) Å and Pd···Pd = 2.31 (3) Å].

### Experimental

All experiments were carried out under an atmosphere of dry nitrogen or argon using Schlenk techniques or in an argon filled glovebox. Solvents ([D_6_]benzene, benzene) were freshly distilled from sodium/benzophenone prior to use. NMR spectra were recorded on a Bruker Avance 400 (^1^H, ^1^H^31^P-COSY) or a Bruker Avance 300 (^31^P, ^31^P{1H}) instrument. Chemical shift values (^1^H) are reported in p.p.m. relative to SiMe_4_ and were referenced to residual solvent signals. The ^31^P{^1^H} and ^31^P NMR chemical shift values were referenced to external H_3_PO_4_ (85%). Abbreviations: d = doublet, t = triplet, m = multiplet, br = broad. Pd(P*t*Bu_3_)_2_ (38 mg, 0.074 mmol) was dissolved in 0.5 ml C_6_D_6_ in a vial. The vial was sealed and stored at room temperature under daylight for six weeks. During this period red plate-shaped crystals formed. Those were collected atop a frit and washed with 2 ml of benzene. Drying *in vacuo* yielded the title compound (8 mg, 0.009 mmol, 24%).

^1^H (400.13 MHz, [D_6_]Benzene, 25°C): δ = 1.56 (m, P*t*Bu_2_), 1.39 (m, HP*t*Bu_2_); ^31^P{^1^H} NMR (121.45 MHz, [D_6_]Benzene, 25°C): δ = 284.3 (t, ^2^*J*_PP_ = 32.2 Hz, P*t*Bu_2_), 57.9 (t, ^2^*J*_PP_ = 32.2 Hz, HP*t*Bu_2_); ^31^P NMR (121.45 MHz, [D_6_]Benzene, 25°C): δ = 284.3 (br, P*t*Bu_2_), 57.9 (d br, ^1^*J*_HP_ = 280 Hz, HP*t*Bu_2_).

### Refinement

H atoms bonded to C were refined using a riding model, with C—H = 0.98 Å and with *U*_iso_(H) = 1.5*U*_eq_(C). The H atom bonded to P was freely refined.

The final difference Fourier map had a peak and a hole in the vicinity of Pd1.

### Crystal data

**Table:**

[Pd_2_(C_8_H_18_P)_2_(C_8_H_19_P)_2_]	*Z* = 1
*M**_r_* = 795.59	*F*(000) = 418
Triclinic, *P*1	*D*_x_ = 1.351 Mg m^−^^3^
Hall symbol: -P 1	Mo *K*α radiation, λ = 0.71073 Å
*a* = 9.0721 (5) Å	Cell parameters from 11232 reflections
*b* = 10.5156 (6) Å	θ = 3.5–28.0°
*c* = 11.5351 (6) Å	µ = 1.10 mm^−^^1^
α = 89.064 (5)°	*T* = 173 K
β = 67.307 (4)°	Plate, red
γ = 75.330 (5)°	0.23 × 0.14 × 0.04 mm
*V* = 978.06 (9) Å^3^	

### Data collection

**Table:**

Stoe IPDS II two-circle diffractometer	4481 independent reflections
Radiation source: fine-focus sealed tube	4083 reflections with *I* > 2σ(*I*)
Graphite monochromator	*R*_int_ = 0.066
ω scans	θ_max_ = 27.5°, θ_min_ = 3.5°
Absorption correction: multi-scan (*MULABS*; Spek, 2009; Blessing, 1995)	*h* = −11→11
*T*_min_ = 0.786, *T*_max_ = 0.957	*k* = −13→13
13922 measured reflections	*l* = −14→14

### Refinement

**Table:**

Refinement on *F*^2^	Primary atom site location: structure-invariant direct methods
Least-squares matrix: full	Secondary atom site location: difference Fourier map
*R*[*F*^2^ > 2σ(*F*^2^)] = 0.042	Hydrogen site location: inferred from neighbouring sites
*wR*(*F*^2^) = 0.111	H atoms treated by a mixture of independent and constrained refinement
*S* = 1.05	*w* = 1/[σ^2^(*F*_o_^2^) + (0.0669*P*)^2^ + 1.1837*P*] where *P* = (*F*_o_^2^ + 2*F*_c_^2^)/3
4481 reflections	(Δ/σ)_max_ < 0.001
176 parameters	Δρ_max_ = 2.31 e Å^−^^3^
0 restraints	Δρ_min_ = −1.34 e Å^−^^3^

### Special details

**Table:**

Geometry. All e.s.d.'s (except the e.s.d. in the dihedral angle between two l.s. planes) are estimated using the full covariance matrix. The cell e.s.d.'s are taken into account individually in the estimation of e.s.d.'s in distances, angles and torsion angles; correlations between e.s.d.'s in cell parameters are only used when they are defined by crystal symmetry. An approximate (isotropic) treatment of cell e.s.d.'s is used for estimating e.s.d.'s involving l.s. planes.
Refinement. Refinement of *F*^2^ against ALL reflections. The weighted *R*-factor *wR* and goodness of fit *S* are based on *F*^2^, conventional *R*-factors *R* are based on *F*, with *F* set to zero for negative *F*^2^. The threshold expression of *F*^2^ > σ(*F*^2^) is used only for calculating *R*-factors(gt) *etc*. and is not relevant to the choice of reflections for refinement. *R*-factors based on *F*^2^ are statistically about twice as large as those based on *F*, and *R*-factors based on ALL data will be even larger.

### Fractional atomic coordinates and isotropic or equivalent isotropic displacement parameters (Å^2^)

**Table:**

	*x*	*y*	*z*	*U*_iso_*/*U*_eq_	
Pd1	0.50058 (3)	0.07733 (2)	0.41089 (2)	0.01892 (10)	
P1	0.46490 (10)	0.14997 (8)	0.61308 (7)	0.02064 (18)	
P2	0.49486 (11)	0.23437 (8)	0.27214 (8)	0.02250 (18)	
H2	0.479 (6)	0.345 (5)	0.312 (4)	0.033 (11)*	
C1	0.2544 (4)	0.2667 (4)	0.7086 (3)	0.0275 (7)	
C2	0.6381 (4)	0.2167 (3)	0.6181 (3)	0.0256 (7)	
C3	0.3122 (5)	0.2678 (4)	0.2244 (3)	0.0305 (7)	
C4	0.6950 (5)	0.2228 (4)	0.1328 (3)	0.0290 (7)	
C11	0.2300 (6)	0.4002 (4)	0.6554 (5)	0.0476 (11)	
H11A	0.3097	0.4445	0.6620	0.071*	
H11B	0.1170	0.4546	0.7034	0.071*	
H11C	0.2476	0.3874	0.5666	0.071*	
C12	0.2212 (6)	0.2872 (5)	0.8483 (4)	0.0439 (10)	
H12A	0.2370	0.2013	0.8827	0.066*	
H12B	0.1070	0.3404	0.8943	0.066*	
H12C	0.2983	0.3328	0.8574	0.066*	
C13	0.1300 (5)	0.1985 (4)	0.6982 (4)	0.0379 (9)	
H13A	0.1452	0.1125	0.7325	0.057*	
H13B	0.1476	0.1859	0.6094	0.057*	
H13C	0.0171	0.2533	0.7461	0.057*	
C21	0.6472 (6)	0.3438 (4)	0.5533 (5)	0.0418 (10)	
H21A	0.6534	0.3300	0.4676	0.063*	
H21B	0.7460	0.3685	0.5496	0.063*	
H21C	0.5480	0.4146	0.6013	0.063*	
C22	0.6292 (6)	0.2379 (6)	0.7517 (4)	0.0456 (11)	
H22A	0.6238	0.1561	0.7931	0.068*	
H22B	0.5299	0.3085	0.8000	0.068*	
H22C	0.7281	0.2626	0.7476	0.068*	
C23	0.7975 (5)	0.1095 (4)	0.5433 (4)	0.0360 (8)	
H23A	0.7947	0.0269	0.5837	0.054*	
H23B	0.8935	0.1373	0.5416	0.054*	
H23C	0.8063	0.0956	0.4568	0.054*	
C31	0.1604 (5)	0.2900 (6)	0.3489 (4)	0.0508 (13)	
H31A	0.1710	0.2125	0.3963	0.076*	
H31B	0.0602	0.3038	0.3315	0.076*	
H31C	0.1526	0.3679	0.3986	0.076*	
C32	0.2917 (7)	0.3882 (6)	0.1519 (5)	0.0564 (14)	
H32A	0.2869	0.4658	0.2005	0.085*	
H32B	0.1889	0.4024	0.1382	0.085*	
H32C	0.3862	0.3741	0.0703	0.085*	
C33	0.3187 (7)	0.1459 (6)	0.1504 (6)	0.0576 (14)	
H33A	0.3317	0.0689	0.1981	0.086*	
H33B	0.4130	0.1307	0.0686	0.086*	
H33C	0.2157	0.1599	0.1370	0.086*	
C41	0.6962 (6)	0.3430 (5)	0.0581 (5)	0.0507 (12)	
H41A	0.6656	0.4223	0.1148	0.076*	
H41B	0.6163	0.3516	0.0186	0.076*	
H41C	0.8074	0.3328	−0.0075	0.076*	
C42	0.8260 (6)	0.2088 (6)	0.1887 (4)	0.0475 (11)	
H42A	0.7977	0.2872	0.2458	0.071*	
H42B	0.9349	0.1998	0.1204	0.071*	
H42C	0.8292	0.1303	0.2356	0.071*	
C43	0.7432 (7)	0.0998 (5)	0.0447 (4)	0.0513 (12)	
H43A	0.7428	0.0222	0.0928	0.077*	
H43B	0.8544	0.0899	−0.0208	0.077*	
H43C	0.6635	0.1081	0.0051	0.077*	

### Atomic displacement parameters (Å^2^)

**Table:**

	*U*^11^	*U*^22^	*U*^33^	*U*^12^	*U*^13^	*U*^23^
Pd1	0.02206 (14)	0.01645 (14)	0.01839 (13)	−0.00473 (9)	−0.00844 (9)	0.00263 (8)
P1	0.0242 (4)	0.0166 (4)	0.0205 (4)	−0.0051 (3)	−0.0085 (3)	0.0012 (3)
P2	0.0298 (4)	0.0190 (4)	0.0194 (4)	−0.0058 (3)	−0.0110 (3)	0.0036 (3)
C1	0.0252 (16)	0.0240 (17)	0.0274 (16)	−0.0013 (13)	−0.0072 (13)	−0.0011 (13)
C2	0.0277 (16)	0.0240 (17)	0.0263 (16)	−0.0077 (13)	−0.0115 (13)	−0.0001 (12)
C3	0.0291 (17)	0.0328 (19)	0.0302 (17)	−0.0051 (15)	−0.0144 (14)	0.0069 (14)
C4	0.0316 (18)	0.0306 (19)	0.0247 (16)	−0.0105 (15)	−0.0098 (14)	0.0054 (13)
C11	0.038 (2)	0.026 (2)	0.062 (3)	0.0038 (17)	−0.010 (2)	0.0070 (19)
C12	0.038 (2)	0.052 (3)	0.0302 (19)	−0.0041 (19)	−0.0060 (17)	−0.0134 (18)
C13	0.0273 (18)	0.040 (2)	0.042 (2)	−0.0073 (16)	−0.0103 (16)	−0.0037 (17)
C21	0.046 (2)	0.030 (2)	0.058 (3)	−0.0197 (18)	−0.025 (2)	0.0115 (18)
C22	0.040 (2)	0.069 (3)	0.035 (2)	−0.018 (2)	−0.0187 (18)	−0.005 (2)
C23	0.0261 (17)	0.034 (2)	0.043 (2)	−0.0082 (15)	−0.0092 (16)	−0.0012 (16)
C31	0.030 (2)	0.078 (4)	0.038 (2)	−0.006 (2)	−0.0127 (18)	0.017 (2)
C32	0.048 (3)	0.060 (3)	0.066 (3)	−0.011 (2)	−0.031 (2)	0.038 (3)
C33	0.061 (3)	0.059 (3)	0.070 (3)	−0.010 (3)	−0.047 (3)	−0.008 (3)
C41	0.043 (2)	0.052 (3)	0.050 (3)	−0.017 (2)	−0.009 (2)	0.027 (2)
C42	0.035 (2)	0.073 (3)	0.037 (2)	−0.019 (2)	−0.0144 (18)	0.009 (2)
C43	0.056 (3)	0.052 (3)	0.033 (2)	−0.013 (2)	−0.004 (2)	−0.0114 (19)

### Geometric parameters (Å, º)

**Table:**

Pd1—P2	2.2860 (8)	C13—H13B	0.9800
Pd1—P1^i^	2.3374 (9)	C13—H13C	0.9800
Pd1—P1	2.3405 (8)	C21—H21A	0.9800
Pd1—Pd1^i^	2.5988 (5)	C21—H21B	0.9800
P1—C2	1.896 (4)	C21—H21C	0.9800
P1—C1	1.898 (4)	C22—H22A	0.9800
P1—Pd1^i^	2.3373 (9)	C22—H22B	0.9800
P2—C4	1.881 (4)	C22—H22C	0.9800
P2—C3	1.891 (4)	C23—H23A	0.9800
P2—H2	1.21 (5)	C23—H23B	0.9800
C1—C11	1.521 (6)	C23—H23C	0.9800
C1—C13	1.523 (5)	C31—H31A	0.9800
C1—C12	1.529 (5)	C31—H31B	0.9800
C2—C21	1.526 (5)	C31—H31C	0.9800
C2—C22	1.529 (5)	C32—H32A	0.9800
C2—C23	1.533 (5)	C32—H32B	0.9800
C3—C32	1.517 (6)	C32—H32C	0.9800
C3—C33	1.528 (6)	C33—H33A	0.9800
C3—C31	1.528 (6)	C33—H33B	0.9800
C4—C41	1.519 (6)	C33—H33C	0.9800
C4—C43	1.520 (6)	C41—H41A	0.9800
C4—C42	1.532 (6)	C41—H41B	0.9800
C11—H11A	0.9800	C41—H41C	0.9800
C11—H11B	0.9800	C42—H42A	0.9800
C11—H11C	0.9800	C42—H42B	0.9800
C12—H12A	0.9800	C42—H42C	0.9800
C12—H12B	0.9800	C43—H43A	0.9800
C12—H12C	0.9800	C43—H43B	0.9800
C13—H13A	0.9800	C43—H43C	0.9800
			
P2—Pd1—P1^i^	130.80 (3)	C1—C13—H13C	109.5
P2—Pd1—P1	116.70 (3)	H13A—C13—H13C	109.5
P1^i^—Pd1—P1	112.50 (2)	H13B—C13—H13C	109.5
P2—Pd1—Pd1^i^	172.88 (3)	C2—C21—H21A	109.5
P1^i^—Pd1—Pd1^i^	56.31 (2)	C2—C21—H21B	109.5
P1—Pd1—Pd1^i^	56.19 (2)	H21A—C21—H21B	109.5
C2—P1—C1	110.95 (16)	C2—C21—H21C	109.5
C2—P1—Pd1^i^	120.70 (11)	H21A—C21—H21C	109.5
C1—P1—Pd1^i^	121.18 (12)	H21B—C21—H21C	109.5
C2—P1—Pd1	114.53 (11)	C2—C22—H22A	109.5
C1—P1—Pd1	114.77 (12)	C2—C22—H22B	109.5
Pd1^i^—P1—Pd1	67.50 (2)	H22A—C22—H22B	109.5
C4—P2—C3	111.79 (16)	C2—C22—H22C	109.5
C4—P2—Pd1	116.35 (12)	H22A—C22—H22C	109.5
C3—P2—Pd1	115.50 (12)	H22B—C22—H22C	109.5
C4—P2—H2	96 (2)	C2—C23—H23A	109.5
C3—P2—H2	99 (2)	C2—C23—H23B	109.5
Pd1—P2—H2	115 (2)	H23A—C23—H23B	109.5
C11—C1—C13	109.2 (3)	C2—C23—H23C	109.5
C11—C1—C12	109.4 (4)	H23A—C23—H23C	109.5
C13—C1—C12	108.0 (3)	H23B—C23—H23C	109.5
C11—C1—P1	112.3 (3)	C3—C31—H31A	109.5
C13—C1—P1	104.7 (3)	C3—C31—H31B	109.5
C12—C1—P1	113.1 (3)	H31A—C31—H31B	109.5
C21—C2—C22	109.5 (3)	C3—C31—H31C	109.5
C21—C2—C23	108.1 (3)	H31A—C31—H31C	109.5
C22—C2—C23	108.3 (3)	H31B—C31—H31C	109.5
C21—C2—P1	112.6 (3)	C3—C32—H32A	109.5
C22—C2—P1	113.2 (3)	C3—C32—H32B	109.5
C23—C2—P1	104.7 (2)	H32A—C32—H32B	109.5
C32—C3—C33	110.2 (4)	C3—C32—H32C	109.5
C32—C3—C31	108.5 (4)	H32A—C32—H32C	109.5
C33—C3—C31	107.5 (4)	H32B—C32—H32C	109.5
C32—C3—P2	115.2 (3)	C3—C33—H33A	109.5
C33—C3—P2	110.3 (3)	C3—C33—H33B	109.5
C31—C3—P2	104.7 (3)	H33A—C33—H33B	109.5
C41—C4—C43	109.4 (4)	C3—C33—H33C	109.5
C41—C4—C42	108.2 (4)	H33A—C33—H33C	109.5
C43—C4—C42	108.0 (4)	H33B—C33—H33C	109.5
C41—C4—P2	114.6 (3)	C4—C41—H41A	109.5
C43—C4—P2	111.1 (3)	C4—C41—H41B	109.5
C42—C4—P2	105.1 (3)	H41A—C41—H41B	109.5
C1—C11—H11A	109.5	C4—C41—H41C	109.5
C1—C11—H11B	109.5	H41A—C41—H41C	109.5
H11A—C11—H11B	109.5	H41B—C41—H41C	109.5
C1—C11—H11C	109.5	C4—C42—H42A	109.5
H11A—C11—H11C	109.5	C4—C42—H42B	109.5
H11B—C11—H11C	109.5	H42A—C42—H42B	109.5
C1—C12—H12A	109.5	C4—C42—H42C	109.5
C1—C12—H12B	109.5	H42A—C42—H42C	109.5
H12A—C12—H12B	109.5	H42B—C42—H42C	109.5
C1—C12—H12C	109.5	C4—C43—H43A	109.5
H12A—C12—H12C	109.5	C4—C43—H43B	109.5
H12B—C12—H12C	109.5	H43A—C43—H43B	109.5
C1—C13—H13A	109.5	C4—C43—H43C	109.5
C1—C13—H13B	109.5	H43A—C43—H43C	109.5
H13A—C13—H13B	109.5	H43B—C43—H43C	109.5
			
P2—Pd1—P1—C2	−65.64 (13)	C1—P1—C2—C21	−65.6 (3)
P1^i^—Pd1—P1—C2	114.74 (13)	Pd1^i^—P1—C2—C21	143.7 (3)
Pd1^i^—Pd1—P1—C2	114.74 (13)	Pd1—P1—C2—C21	66.4 (3)
P2—Pd1—P1—C1	64.42 (13)	C1—P1—C2—C22	59.4 (3)
P1^i^—Pd1—P1—C1	−115.21 (13)	Pd1^i^—P1—C2—C22	−91.3 (3)
Pd1^i^—Pd1—P1—C1	−115.21 (13)	Pd1—P1—C2—C22	−168.7 (3)
P2—Pd1—P1—Pd1^i^	179.63 (3)	C1—P1—C2—C23	177.2 (2)
P1^i^—Pd1—P1—Pd1^i^	0.0	Pd1^i^—P1—C2—C23	26.5 (3)
P1^i^—Pd1—P2—C4	−67.41 (14)	Pd1—P1—C2—C23	−50.9 (3)
P1—Pd1—P2—C4	113.05 (14)	C4—P2—C3—C32	−54.6 (4)
P1^i^—Pd1—P2—C3	66.64 (15)	Pd1—P2—C3—C32	169.3 (3)
P1—Pd1—P2—C3	−112.90 (14)	C4—P2—C3—C33	71.0 (4)
C2—P1—C1—C11	63.1 (3)	Pd1—P2—C3—C33	−65.1 (4)
Pd1^i^—P1—C1—C11	−146.4 (3)	C4—P2—C3—C31	−173.6 (3)
Pd1—P1—C1—C11	−68.7 (3)	Pd1—P2—C3—C31	50.3 (3)
C2—P1—C1—C13	−178.6 (2)	C3—P2—C4—C41	56.2 (4)
Pd1^i^—P1—C1—C13	−28.1 (3)	Pd1—P2—C4—C41	−168.2 (3)
Pd1—P1—C1—C13	49.6 (3)	C3—P2—C4—C43	−68.6 (4)
C2—P1—C1—C12	−61.3 (3)	Pd1—P2—C4—C43	67.1 (3)
Pd1^i^—P1—C1—C12	89.3 (3)	C3—P2—C4—C42	174.9 (3)
Pd1—P1—C1—C12	167.0 (3)	Pd1—P2—C4—C42	−49.5 (3)

Symmetry code: (i) −*x*+1, −*y*, −*z*+1.
